# Data-Led Suzuki-Miyaura Reaction Optimization: Development of a Short Course for Postgraduate Synthetic Chemists

**DOI:** 10.1021/acs.jchemed.4c01194

**Published:** 2025-01-22

**Authors:** Stuart C. Smith, Barnabas A. Franklin, Christopher S. Horbaczewskyj, James D. D’Souza Metcalf, Jacob J. Walder, Peter O’Brien, Ian J. S. Fairlamb

**Affiliations:** Department of Chemistry, University of York, Heslington, York YO10 5DD, United Kingdom

**Keywords:** Graduate Education/Research, Organic Chemistry, Workshop, Interdisciplinary, Catalysis

## Abstract

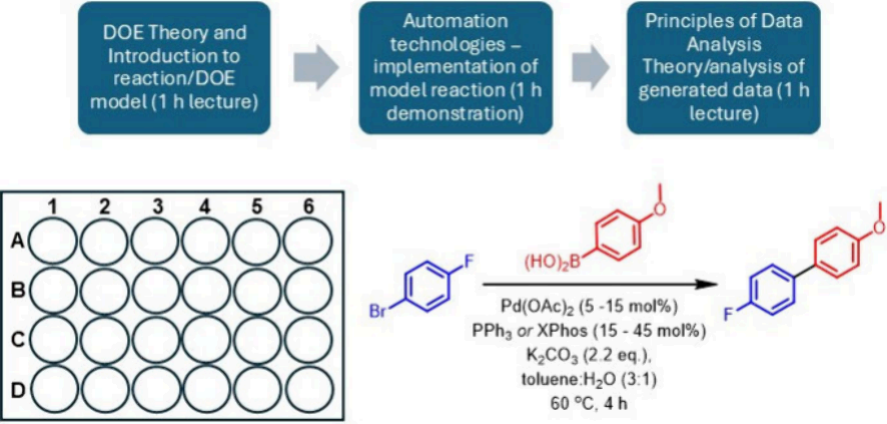

A two-day workshop activity is described in which postgraduate students are introduced to (i) the theory and application of Design-of-Experiments (DOE) approaches and (ii) the implementation of affordable automation technologies and related data analysis of a system of catalytic interest. This work involved the design and delivery of a short lecture to introduce the theory of DOE followed by practical demonstrations of the application of automation technologies. Specifically, a fractional factorial design was used to interrogate the input space—base, solvent, temperature, time—of the Suzuki-Miyaura cross-coupling (SMCC) of *para*-bromoanisole and *para*-fluorophenylboronic acid using automated solid and liquid handling robots and online HPLC analysis. This was supplemented by a second lecture following data acquisition in which the collected HPLC data was analyzed. The workshop was delivered to a cohort of 15 students at the postgraduate level. Pleasingly, students demonstrated a high degree of engagement with this course structure and reported an increased theoretical understanding of DOE approaches to reaction optimization.

## Introduction

Graduates in the chemical sciences are currently experiencing a significant skills and knowledge gap amid the evolving requirement for understanding of automation technologies and data science approaches to reaction optimization and development.^1^ Automation technologies are revolutionizing the development of organic and catalytic processes in academic and industrial settings by greatly increasing the number of experimental variables that can be rapidly and efficiently assayed in reaction optimization and discovery assays.^1a,2^ There is strong evidence that the future of synthetic chemistry will require an awareness of high-throughput experimentation (HTE) approaches and the employment of data science methods and tools to effectively contribute to modern chemical sciences. Statistically centered approaches—including Design of Experiments (DOE)—are highly complementary to these technologies and methods.^3^ Despite the increasing importance of HTE, statistical science and DOE techniques and technologies to academia and industry, few undergraduate and postgraduate curricula have evolved to integrate this knowledge base.^4−6^ There are limited reports of such approaches having been integrated into an undergraduate chemistry degree curriculum which results in many graduates having minimal experience in this growing field and lacking the skills needed to apply these techniques and tools.^7−9^ With the exception of specialist doctoral training centers, a similar issue exists in postgraduate academic settings, where high cost-barriers and a lack of expertise often prevent the deployment and development of this emerging skill-need. Following the development of an undergraduate-level course to address this critical issue (see the Supporting Information), we found that insufficient scaffolding in automation technologies and data-handling were major barriers to students at that present level. To address this issue, we reformulated the course presented herein for delivery to postgraduate students (whose additional laboratory experience was envisaged to lower the barrier to access reported for undergraduate students).

Efficient reaction optimization is challenging due to both the large number of variables which might be investigated (reagents, catalysts, solvents, temperatures, stirring rates, *etc*.) as well as the potential for interactions between these variables. Traditional One-Factor-at-at-Time (OFAT) approaches to reaction optimization typically involve identification of a model reaction and sequential optimization of each process variable in-turn. This is both resource-inefficient (since the entire chemical space must be assayed to ensure that optimal OFAT conditions are identified) and risks failure to identify true optimal conditions for a given transformation.

DOE is a statistical approach to reaction optimization that seeks to address these challenges by sampling a statistically defined subspace of the target chemical space. Measurement of target variables of interest (*e.g*., conversion to product) at defined points of this multidimensional subspace allows development of models which can be interpolated (*i.e*., to generate a predictive hypersurface of all input variables). Statistical DOE approaches were first formulated by Fisher in 1935^10^ but have since been widely adopted in the field of chemical reaction optimization.^11−13^

The work outlined herein aims to address this issue through the design and delivery of an easily implemented postgraduate course focused on training students in the analysis of a Suzuki-Miyaura cross-coupling (SMCC) reaction, which has been selected as it is one of the most applied transition metal-catalyzed reactions in applied chemical industries and diverse academic fields.^14−16^ The emphasis of the training is on the use of industry-standard DOE software (MODDE), usage of a Chemspeed robotic platform and solid dispenser and analysis of the resulting data set. We believe that the presented workshop is both hardware and software agnostic and can be readily adapted to a variety of formats, *e.g*., with small tutorial groups, one-to-one training or without access to a robotic platform.

## Pedagogical Goals

Several pedagogical goals were developed for this activity prior to its implementation and were formulated as learning outcomes. These outcomes were useful to communicate the goals of the training activities to learners. Additionally, these outcomes served as a benchmark against which the activity could be evaluated following implementation. In particular, the in-house developed questionnaire administered to students before and after the course could be tailored to these goals. This feedback was the major evaluative mechanism for the presented activity.

- Develop an understanding of the principles of chemical applications of DOE.
- Demonstrate to students how to define an optimization input space and how to create a DOE experimental matrix.
- Develop an appreciation for how students could apply available automation tools to their own research.
- Demonstrate the application of data analysis principles to a data set produced via a DOE-led reaction optimization campaign.

## Overview of Course

**Figure 1:**
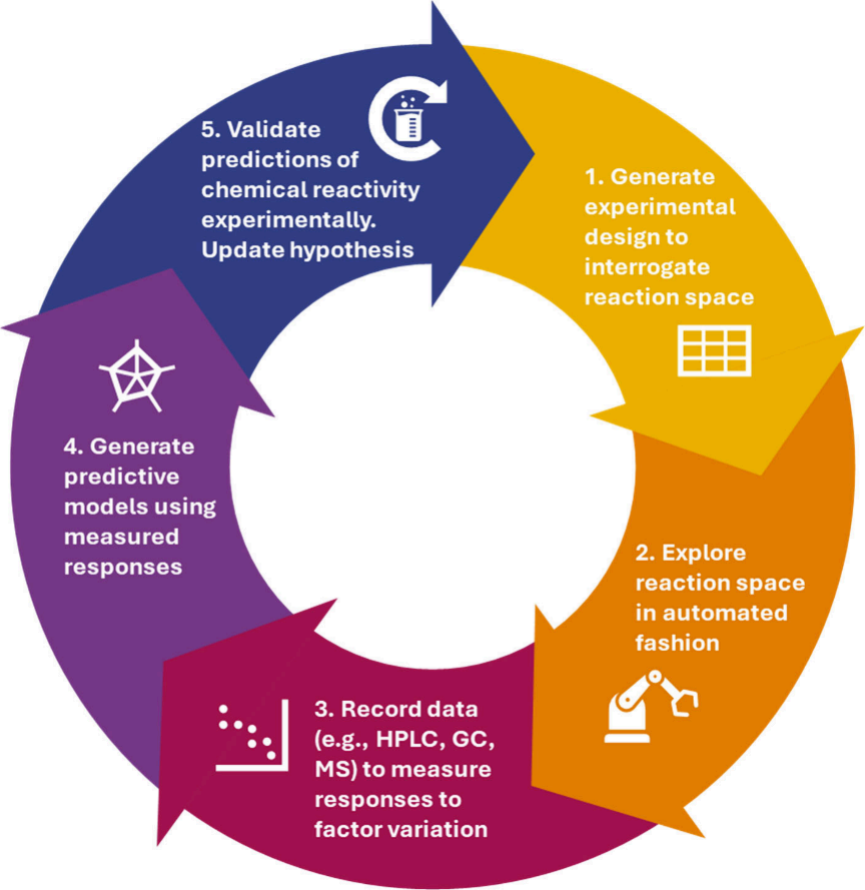
Summary of the experimental workflow developed during this activity.

This activity was designed as an optional course for PhD-level postgraduate students undertaking research in synthetic organic chemistry laboratories and was not formally graded. Students enroll onto the course based on their planned research activities (*i.e*., whether the course is likely to be beneficial to their studies, and whether they can balance the necessary time commitment with other responsibilities, *e.g*., experimental laboratory work, administrative tasks, data analysis, *etc*.). First, students were introduced to the concepts of DOE in a short lecture (1 h). Next, a demonstration of a solid-handling robot and a liquid-handling platform (Chemspeed Technologies systems) was made (1 h). The reactions that were set up during this demonstration were allowed to proceed for 18 h. HPLC data obtained from this activity were processed, and the data set was analyzed in a workshop-style format with the students (1 h). In total, the course took approximately 3 h (total engagment time for students) to complete. The overall workflow is summarized in Figure 1.

## Experiment

**Scheme 1:**
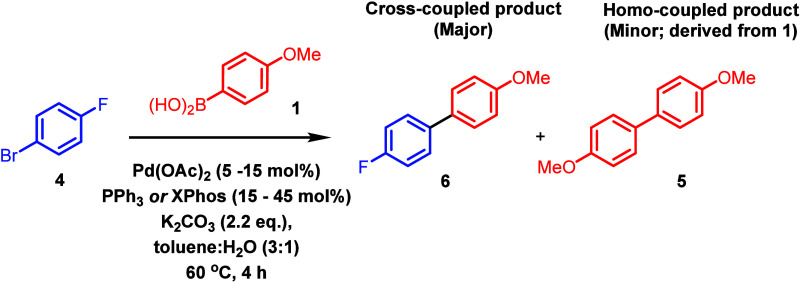
SMCC Reaction Space of *para*-Bromofluorobenzene **4** and with *para*-Methoxyphenylboronic Acid **1** to Give Cross-Coupled Product **6** (Major, Desired Product) and Homo-Coupled Product **5** (Minor Side-Product) Note: The compound numbering follows the order in which each appears in the HPLC chromatograms (see Figure 3*vide infra*). All executed experiments are described in the Supporting Information. The procedure employed is based on that reported by Niwa and co-workers (with modification for the DOE workflow).^17^

All reagents were sourced commercially and used as received without further purification. All solvents were sparged via a cannula with nitrogen prior to use. The procedure was adapted from a literature-reported^13^ SMCC reaction (Scheme 1).

**Figure 2:**
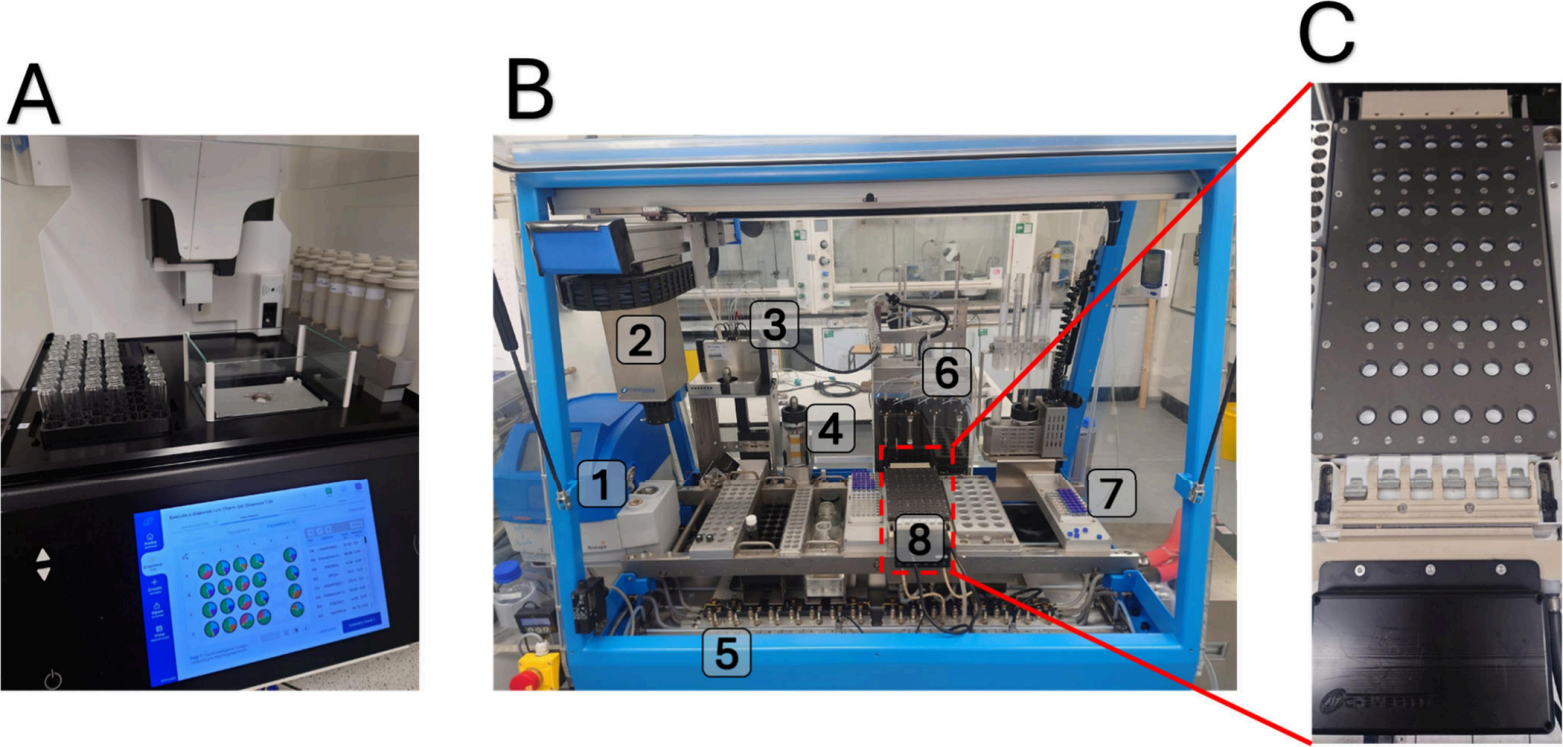
Automation equipment was employed during execution of demonstration experiments.

All reactions were carried out in 20 individual 8 mL vials into which Pd(OAc)_2_ (5, 10, or 15 mol %), ligand (PPh_3_, XPhos, SPhos or Xantphos – 15, 30, or 45 mol %), K_2_CO_3_ (60.8 mg, 0.44 mmol, 2.2 equiv), and aryl boronic acid (33.4 mg, 0.22 mmol, 1.1 equiv) were dispensed using a Chemspeed Crystal Powder Dose solid-dispensing unit. Each vial was placed inside a Chemspeed ISYNTH robotic platform, and the platform's liquid handling unit dispensed a stock solution of toluene (1.0 mL) containing trimethoxybenzene (16.8 mg, 0.1 mmol, 0.5 equiv; internal standard), the aryl bromide (35 mg, 0.2 mmol, 1.0 equiv) and H_2_O (1.0 mL). The vials were then heated to 60 °C and shaken for 18 h. After this time, the aqueous and organic layers were allowed to settle before 50 μL of the organic layer of each reaction mixture was taken and dispensed into a GC vial, which was then diluted with MeCN. For each diluted reaction mixture, HPLC analysis was performed on an isocratic gradient of MeCN:H_2_O (50:50, *v/v*), with 0.1% TFA. HPLC peak areas were quantified using calibration curves to give the conversion to product for each of the reactions. Chromatograms show all reaction components and the internal standard (*vide infra*). These results were then inputted into MODDE software and contour plots were generated. The automated apparatus used in this experiment is shown in Figure 2 (note: it is also possible to use a multiposition standard reaction carousel apparatus for this DOE study, the details of which can be found in the Supporting Information).

Panel A of Figure 2 shows a Chemspeed PowderDose Solid Handling Robot. The system is interfaced with a touchscreen for operation but may also be operated with a mouse/keyboard. Solids are loaded into beige dispensers that dispense via a granular screw mechanism. Dispensers are labeled with Radiofrequency ID (RFID) labels to allow automated identification of the contents. Vials are loaded into up to three 24-well plates. The robotic arm automates movement of plates to the balance pan and movement of the dispenser. Solids are then dispensed with a predefined tolerance.

Panel B of Figure 2 shows a Chemspeed ISYNTH automated synthesis platform. This platform is highly modular and configurable and includes: a microwave reactor (**1**); a robotic arm on XYZA gantry (**2**); an attachment for robotic arm with four needles for solution transfer (**3**); an attachment for robotic arm to allow drawer movement on ISYNTH reactor (**4**); connections to inert gas, power, and temperature control (**5**); syringe drivers for liquid handling (**6**); a module for automated HPLC sample preparation (**7**), and an ISYNTH 48-well parallel screening synthesis plate (**8**).

Panel C of Figure 2 shows a magnified image of the ISYNTH 48-well parallel screening synthesis plate. Sliding drawers on each vertical column are used to control connections to the vacuum and inert gas. Heating/cooling is achieved with a Huber temperature control unit and stirring is achieved via circular agitation.

## Results and Discussion

**Figure 3:**
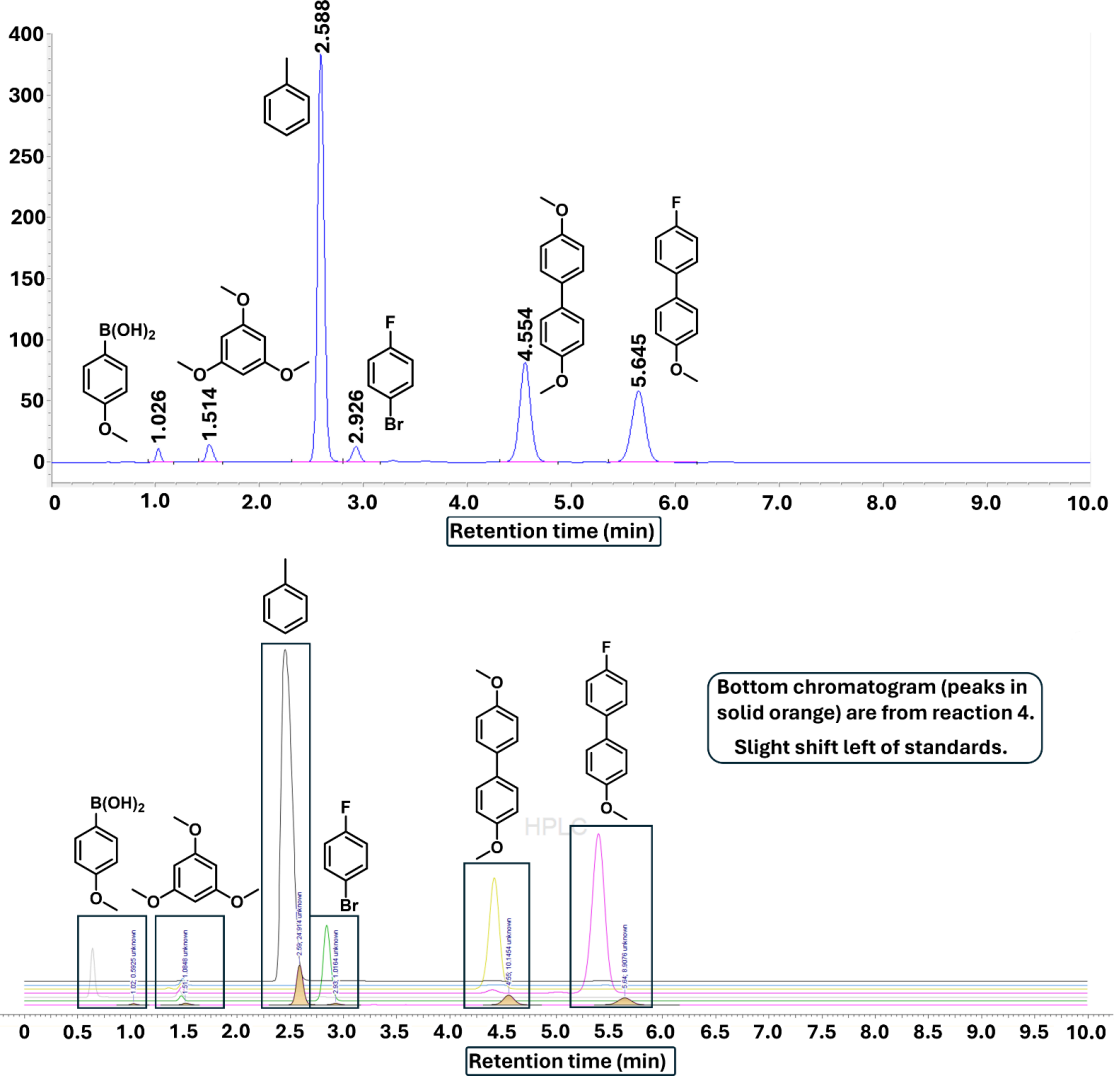
HPLC trace of mixture of 50 mM of each standard and assignments (upper). Comparison of assigned standards and representative reaction mixture (lower). Isocratic gradient of MeCN:H_2_O (50:50, *v/v*), 0.1% TFA over 10 min, and 0.5 mL min^–1^ flow rate. (Note: A GC-FID assay was found to be incompatible with this particular SMCC reaction.)

An HPLC assay was developed to allow for rapid analysis of the reaction mixture. Analytical standards were prepared and analyzed, including *para*-anisoleboronic acid (**1**, starting material (SM)), trimethoxybenzene (**2**, internal standard), toluene (**3**, solvent), *para*-bromofluorobenzene (**4**, SM), 4,4′-*para*-dimethoxybiphenyl (**5**, homocoupled product of the arylboronic acid), and 4-fluoro-4′-methoxy-1,1′-biphenyl (**6**, product). Exemplar chromatograms are shown in Figure 3, including a typical reaction mixture.

**Figure 4:**
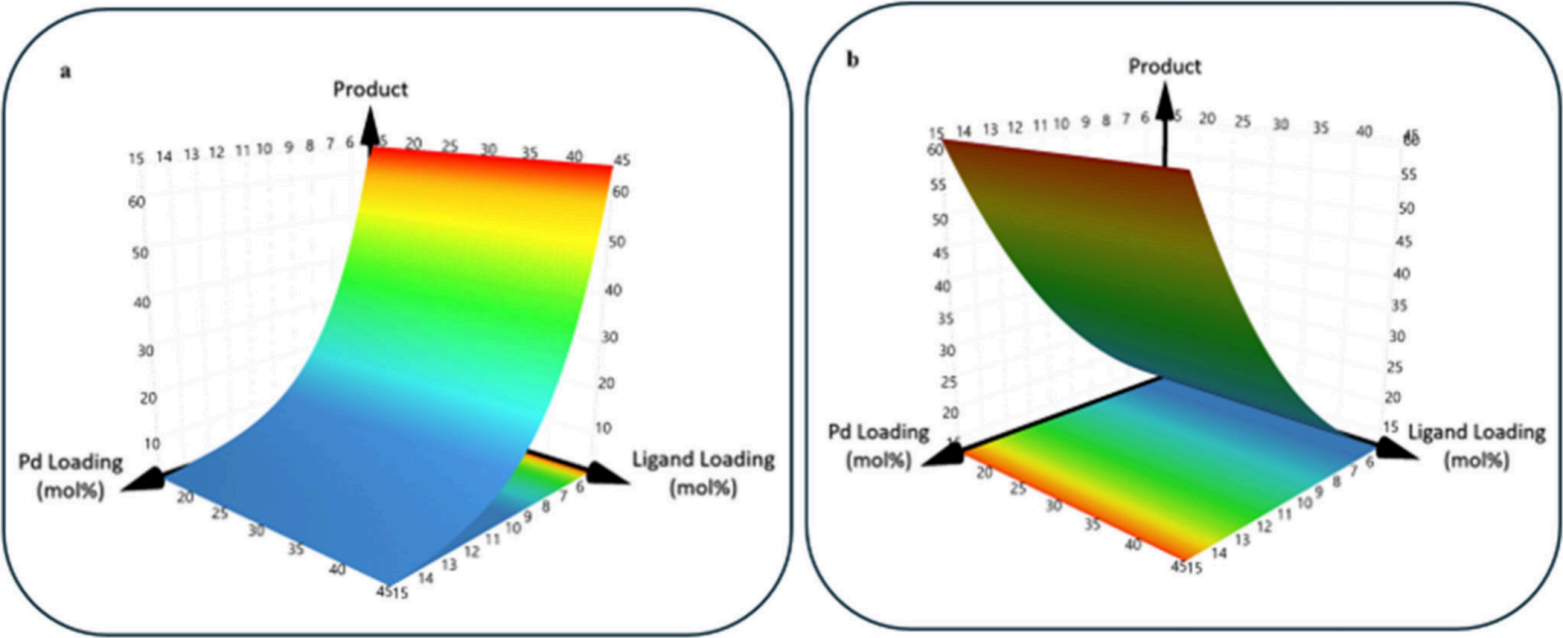
Contour response surfaces for product conversion (%) from the automated DOE-led analysis of SMCC input space. Plot **4a** shows the variation of product conversion with palladium loading (5–15 mol %) and triphenylphosphine loading (15–45 mol %). Plot **4b** shows the variation of product conversion with palladium loading (5–15 mol %) and XPhos loading (15–45 mol %).

**Figure 5:**
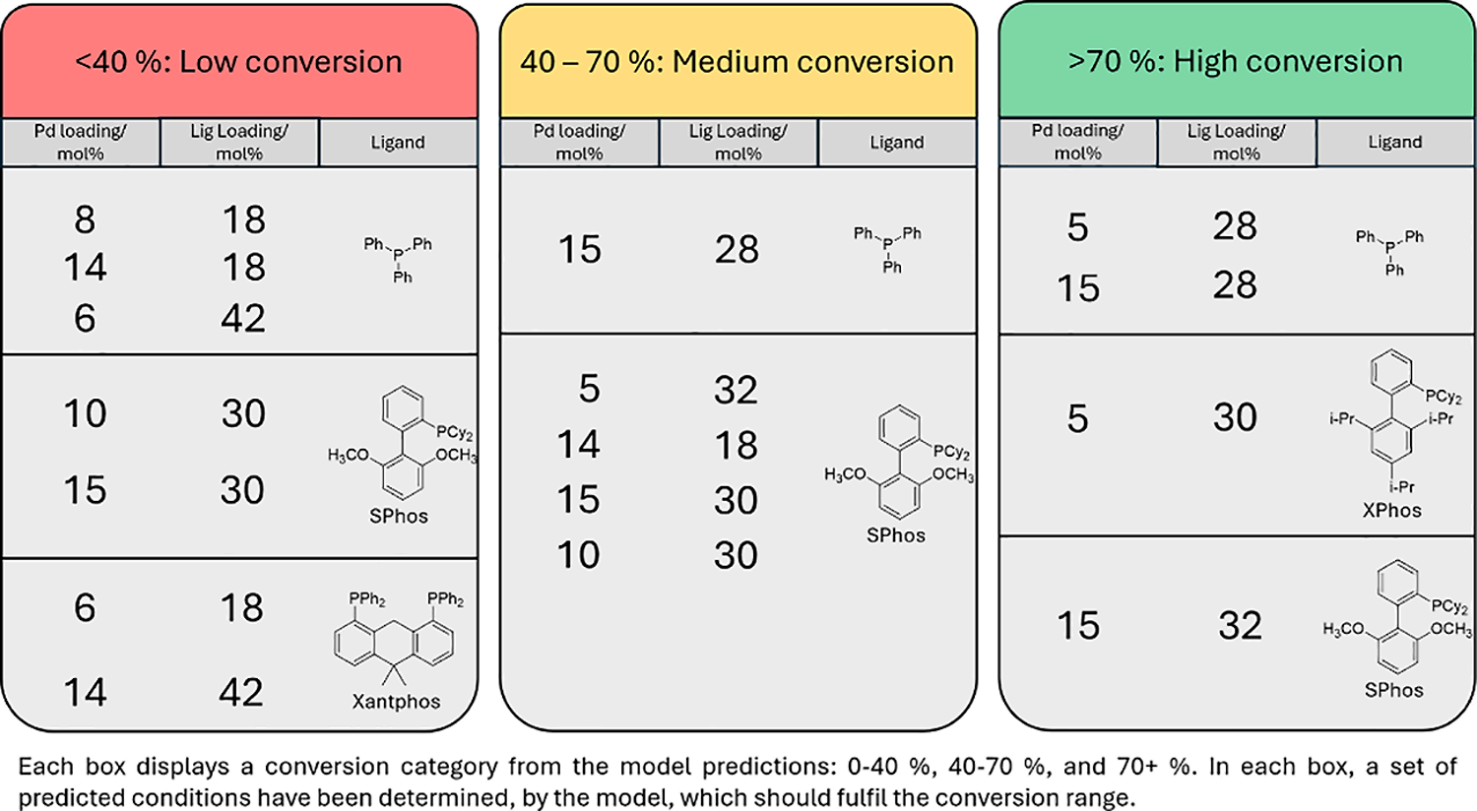
Key reaction outcome data from the DoE study (reaction described in Scheme 1).

Following execution of the DOE-led investigation, HPLC data were recorded using the Chemspeed SWING platform and an Agilent HPLC instrument (using the method described above) (data for 40 HPLC chromatograms can be found in the Supporting Information). The resulting peak areas were input into the software, MODDE, to generate a range of output surfaces, with product conversion as the major response recorded. Exemplar plots from this analysis are shown in Figure 4, and all additional analysis is recorded in the Supporting Information. The key data from the investigation are further summarized in Figure 5 to give a feel for the reaction performance, moving from low to medium to high product conversions.

It is interesting to note the distinct variation in conversion response (indicated on the *z*-axis of each plot) for the different ligands presented in plots **4a** and **4b**. Higher product conversions are observed for higher palladium loadings in the case of triphenylphosphine, with little influence observed from ligand loading. While, in contrast, lower loadings of palladium are preferred for couplings involving XPhos (indicating possible divergence in a catalytic mechanism). These nuances were highlighted and formed the basis of discussion with the students in the final lecture, where it was emphasized that results such as these would warrant further exploration.

### Workshop Activity

A cohort of 15 PhD students attended the workshop (who had voluntarily responded to a department-circulated advertisement for the training). Students were asked before attending the workshop to complete an anonymized, in-house developed questionnaire in which they responded numerically [**1** – Strongly Disagree; **5** – Strongly Agree] to the following competency statements:

A. “I am confident in the practical application of DOE techniques to reaction optimization.”
B. “I am familiar with the theoretical basis for DOE approaches.”
C. “I feel confident in my ability to design and execute a DOE-led reaction optimization campaign.”
D. “I understand how I could use available automation tools for reaction optimization.”
E. “I understand how to analyze the output from a DOE-led reaction optimization campaign.”

**Figure 6:**
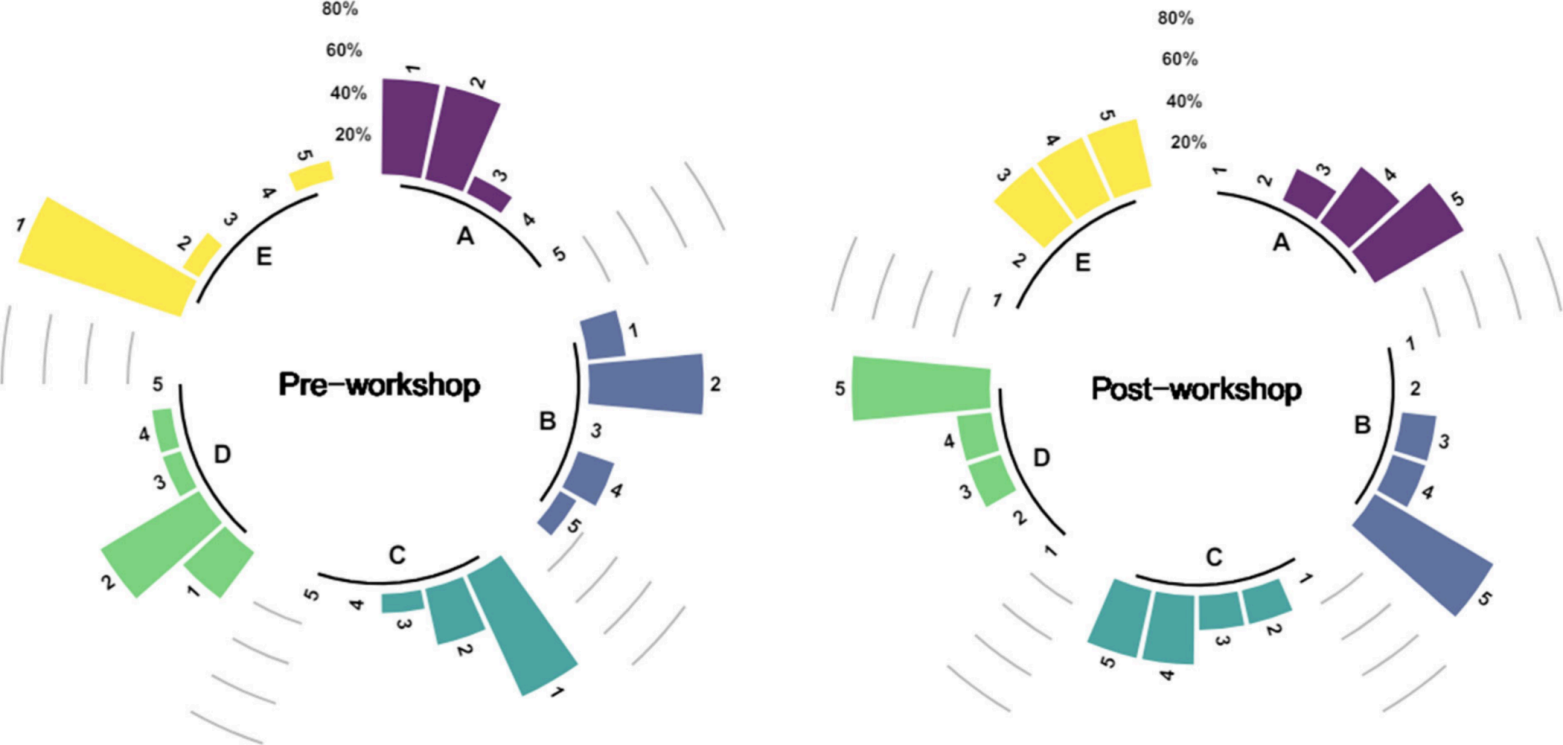
Graphical representation of learner (*N* = 15) responses to a survey before (left) and after (right) delivery of the course described in this work. Levels of response: **1** – Strongly disagree; **2** – Disagree; **3**- Neither agree nor disagree; **4** – Agree; **5** – Strongly agree. Questions were presented in randomized order, and all responses were anonymized.

The results of this survey are listed in Figure 6. Overall, students reported a low level of familiarity and confidence with DOE theory and application, with some learners reporting higher levels of familiarity with automation tools. The survey was readministered following delivery of the course. Pleasingly, the results of the postworkshop questionnaire showed that the confidence of students had significantly increased in their ratings of all competency statements where 80% of students reported that they were familiar with the theoretical basis of DOE, the practical application of DOE and how they could use automation tools. The lowest overall confidence was reported in learners’ ability to analyze the output from a DOE-led reaction optimization campaign.

The results of this survey suggested to us that the design and delivery of this course were sufficient to broadly meet the pedagogical goals set for this work (with the possible exception of demonstrating the application of data analysis principles produced via a DOE-led reaction optimization campaign). This suggests to us that future implementations of this course will benefit from increased emphasis on this aspect in the postdemonstration supporting lecture. In particular, it is intended that a practice data set will be made available to students prior to attending this lecture with short, instructional commentaries on how it may be analyzed. In combination with the Supporting Information (including instructional videos) which were produced following this course (see the Supporting Information), it is anticipated that students will become more confident in analyzing the data sets produced from these activities. In terms of general applicability, it should be noted that we believe that the workflow developed in this work is both hardware and software agnostic, and we anticipate that this work may be implemented in a variety of forms without erosion of the learning objectives. It is important to note that the workshop activity can be conducted using a small carousel screening platform (e.g., using low-cost commercial products) *in lieu* of a high-end robotic system as described in this work. DOE designs can be translated directly into Excel spreadsheets, and the reactions can even be executed one-at-a-time in round-bottomed flasks (if necessary) without hindering delivery of the pedagogical goals.

## Conclusion

A course^i^ has been designed and implemented which successfully introduced DOE and automation approaches to organic reaction optimization within the postgraduate chemistry curriculum. It has been demonstrated that the pedagogical goals developed to support this course were met through formalized written student feedback before and after the delivery of the course. We were particularly pleased with the reported confidence that students felt in applying the concepts explored in the course to their own research. The work adds to training for postgraduates^18^ working with popular Pd-catalyzed cross-coupling reactions.^19−21^ We hope that the community will appreciate this resource as a simple and effective method by which to introduce students to critical concepts in HTE and data analysis and thereby empower them to effectively participate in the evolving workplace of the chemical sciences.

## Supporting Information Available

Methodology used and synthetic chemistry details (experimental, including exemplar NMR spectral data), information concerning equipment, safety risk assessments and initial course developments underpinning the activity described in the main article (PDF)

## Footnotes

i We have included details of our preliminary course developments underpinning this activity in the Supporting Information.
