# Supplementary material for: Data-Led Suzuki-Miyaura Reaction Optimization: Development of a Short Course for Postgraduate Synthetic Chemists
Source: J Chem Educ. 2025 Jan 22;102(2):697–703. doi: 10.1021/acs.jchemed.4c01194 (PMC11823413; doi:10.1021/acs.jchemed.4c01194)
Supplement: Supplementary file 1 — ed4c01194_si_001.pdf [file ed4c01194_si_001.pdf]

Supporting Information

Data-led Suzuki-Miyaura Reaction Optimization: Development of a Short Course for Postgraduate Synthetic Chemists

Stuart C. Smith, Barnabas A. Franklin, Christopher S. Horbaczewskyj, James D. D'Souza Metcalf, Jacob J. Walder, Peter O'Brien, Ian J. S. Fairlamb*

Department of Chemistry, University of York, York, YO10 5DD, UK.

ian.fairlamb@york.ac.uk

Table of Contents

Experimental **1**
List of Equipment **5**
Risk Assessment **10**
Additional Files **25**
Initial Course Developments (footnote i, main manuscript) **26**

Experimental

All reagents were sourced commercially and used as received without purification, with the exception of the cross-coupled product (**6**) which was independently prepared *via* a literature-reported Suzuki-Miyaura cross-coupling.[1] Palladium acetate, 4,4'-*para*-difluorobiphenyl, $Ba(OH)_2.8H_2O$, and 4,4'-*para*-dimethoxybiphenyl were sourced from Sigma Aldrich. *Para*-bromofluorobenzene and *para*-anisoleboronic acid were sourced from Apollo Scientific. Triphenylphosphine was sourced from ThermoScientific. Fluorobenzene was sourced from Alfa Aesar.

All solvents were used as received from Fisher Scientific, with the exception of THF in the preparation of **6** which was dried in a Solvent Purification System and stored over 4 Å molecular sieves.

4-Fluoro-4'-methoxy-1,1'-biphenyl 6

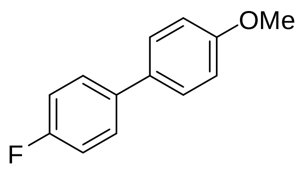

6

4-Fluoro-1-bromobenzene (0.16 mL, 1.50 mmol, 1.0 eq.) was added to a stirred solution of 4-methoxyphenyl boronic acid (274 mg, 1.80 mmol, 1.2 eq.), $Pd(OAc)_2$ (8.4 mg, 0.038 mmol, 0.025 eq.), PPh_3 (39.3 mg, 0.15 mmol, 0.1 eq.), and $Ba(OH)_2{\cdot}8H_2O$ (946 mg, 3.0 mmol, 2.0 eq.) in THF/H_2O (3.6 mL, 5:1) in an oven-dried Schlenk tube under N_2. The Schlenk tube was fitted with a cold finger and the reaction mixture was stirred at 80 °C for 18 h. After this time, the mixture was cooled to rt, and H_2O (5 mL) was added, and the organic layer formed was washed with Et_2O (3 x 10 mL). The combined organic layer was washed with brine (5 mL), dried ($MgSO_4$), filtered, and concentrated to give the crude product. Purification by silica plug using 95:5 Pet-ether-EtOAc as eluent gave the biphenyl product as a white solid (300 mg, 99% yield), mp = 93 – 94 °C; R_F (95:5 Pet-ether-EtOAc) 0.31; IR (ATR) 3020, 2964, 2841, 1607, 1497, 1291, 1238, 1039, 826, 511 cm^{-1}; ^1H NMR (400 MHz, $CDCl_3$) δ 7.53 – 7.46 (m, 4H, Ar), 7.15 – 7.07 (m, 2H, Ar), 7.01 – 6.95 (m, 2H, Ar), 3.86 (s, 3H, OCH_3); ^{13}C NMR (101 MHz, $CDCl_3$) δ 162.2 (d, J = 245.5 Hz, **C**F), 159.2 (**C**OCH_3), 137.1 (d, J = 3.5 Hz, **C**), 133.0 (**C**), 128.4 (d, J = 8.0 Hz, Ar), 128.2 (Ar), 115.7 (d, J = 21.0 Hz, Ar), 114.4 (Ar), 55.5 (O**C**H_3); ^{19}F (376 MHz, $CDCl_3$) δ –116.7 (t, J = 6.5 Hz, C**F**); HRMS (EI) m/z calcd for $C_{13}H_{11}OF$ (M) 202.0788, found 202.0783 (–2.7 ppm difference). Spectroscopic data is consistent with that reported in the literature.[1]

Lab book reference: JJW-3-38

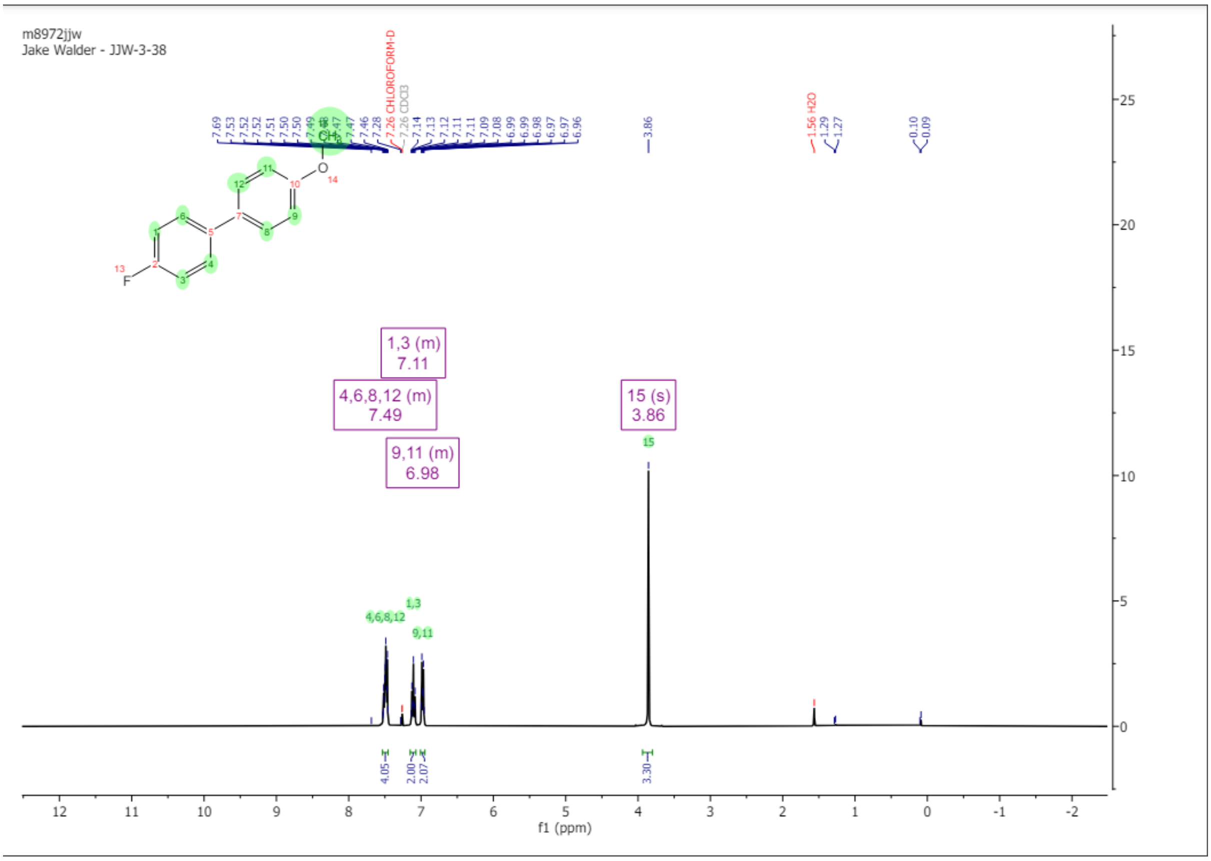

Figure S1: ^{1}H NMR ($CDCl_3$, 400 MHz, 298 K) spectrum of **6**.

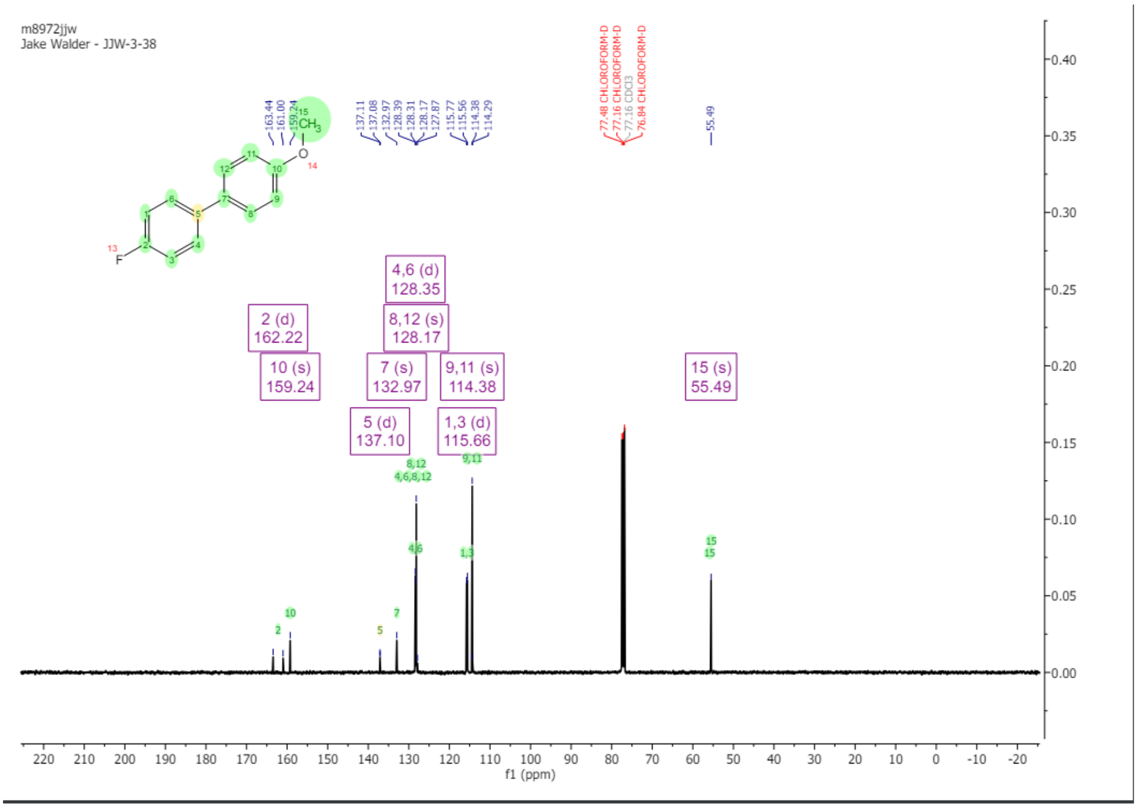

Figure S2: ^{13}C NMR ($CDCl_3$, 100 MHz, 298 K) spectrum of **6**.

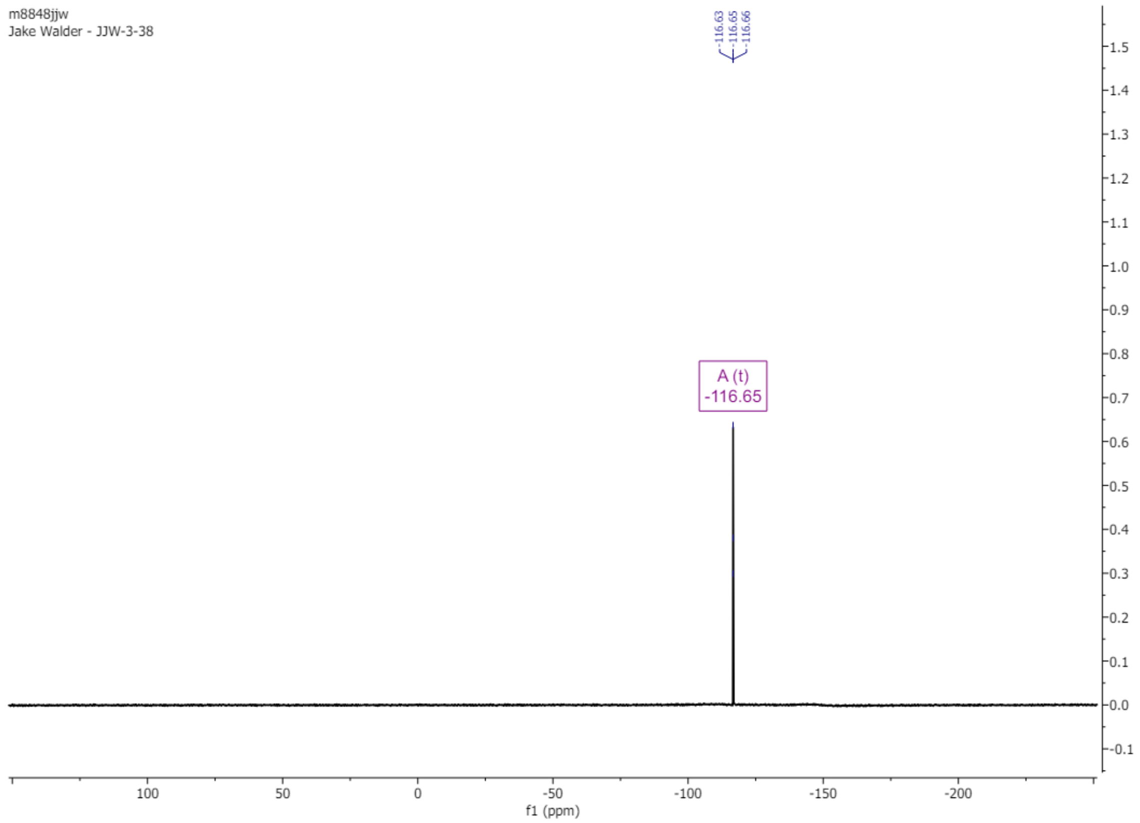

Figure S3: ^{19}F NMR ($CDCl_3$, 376 MHz, 298 K) spectrum of **6**.

List of Equipment

Chemicals	Quantity / mg	CAS
Para-bromofluorobenzene	1260	460-00-4
Para-methoxyphenylboronic acid	1203.48	5720-07-0
Palladium (II) acetate	166.14	3375-31-3
SPhos	270.95	657408-07-6
XPhos	171.62	564483-18-7
Triphenylphosphine	157.38	603-35-0
Xantphos	347.17	161265-03-8
Potassium carbonate	60.81	584-08-7
1,3,5-trimethoxybenzene	16.82	621-23-8
**Solvents**		
Toluene	54	108-88-3
Water	18	
**Access needed to the following**		
Top-pan balance		
Chemspeed SWING automated synthesis platform*		
Chemspeed PowerDose Solid Handling Robot**		
(NMR)		
(HPLC)**		
**Consumables**		
Weighing paper		
Disposable needles		
Disposable syringes (various volumes)		
8 mL vials and septa		
**Waste**		
Flammable solvent		
Aqueous waste		
Sharps bins		
Offensive waste		

Figure S4: List of equipment necessary to execute the experimental work in this manuscript.

* The automation of this synthesis could be readily adapted to be performed on a Radley's carousel or a heating block adapted to run multiple reactions simultaneously. In this workflow, solids and magnetic flea would be charged to each reaction vessel. Each vessel would be set inert under dynamic nitrogen purge for 15 min and sealed. Liquid reagents and solvents would be added *via* syringe and reactions would be heated with water cooling for the reaction duration. The setup for a Radley's carousel is shown in **Figure S6**.

** Manual weighing on an analytical balance could be easily implemented in place of an automated platform.

On a Radley's carousel, each reaction vessel should be equipped with a magnetic stirrer flea, and the required amounts of solid reagents ($Pd(OAc)_2$, ligand, potassium carbonate, 1,3,5-trimethoxybenzene, and *para*-methoxyphenyloboronic acid) weighed out and added to the vessels. The vessels are then placed on the carousel, which is itself placed on a heating block. To create an inert atmosphere, the carousel should be connected to a nitrogen line using a central inlet. The lids of all vessels should be opened parallel to the gas flow to allow for a dynamic nitrogen purge, which should be performed for 15 minutes. After purging, the lids should be closed perpendicular to the gas flow and sealed with septa.

Following this, the specified amounts of liquid reagents (*para*-bromofluorobenzene) and solvent (water and toluene) should be injected into each vessel through the septa using a calibrated syringe. The reaction setup is then heated to 80 °C, with water cooling in place. The reaction mixtures should be stirred for the specified duration, usually 4 hours, after which the vessels are allowed to cool to room temperature before opening. Upon completion of the reaction, the vessels should be quenched with saturated ammonium chloride solution. The organic layer should be extracted and the sample analysed. with an LC system as described in the main manuscript.

The synthesis can also be adapted for use in reaction vials, offering a low-cost and straightforward method for conducting multiple reactions in parallel. Each reaction vial should be equipped with a magnetic stirrer flea, and the required amounts of solid reagents ($Pd(OAc)_2$, ligand, potassium carbonate, 1,35-trimethoxybenzene, and *para*-methoxyphenylboronic acid) should be weighed out and added to the vials. Once prepared, the vials should be sealed with crimped or screw-top caps fitted with a PTFE/silicone septum.

To create an inert atmosphere, a needle connected to a nitrogen line should be inserted through the septum, allowing for a slow purge of nitrogen gas. Simultaneously, a second needle should be inserted to act as a vent. The purge should continue for 10–15 minutes to ensure an oxygen-free environment. Following this, the vent needle should be removed, leaving the nitrogen inlet in place.

Liquid reagents and solvent should be added to each vial using a calibrated syringe, piercing the septum to introduce the correct volumes. The vials should then be placed on a heating block or a stirring hot plate with individual slots for parallel heating. The temperature should be set to the required reaction conditions, typically 80 °C, and the reaction mixtures stirred at the set temperature for the specified duration, usually 4 hours.

After the reaction, the vials should be allowed to cool to room temperature. The septa can then be removed, and the reaction mixtures quenched with saturated ammonium chloride solution. The organic layer should be extracted and the sample analysed with an LC system as described in the main manuscript.

Finally, the synthesis could alternatively be performed in round-bottomed flasks, which provide flexibility for larger-scale reactions if desired. Each round-bottomed flask should be equipped with a magnetic stirrer flea and charged with the required amounts of solid reagents (($Pd(OAc)_2$, ligand, potassium carbonate, 1,35-trimethoxybenzene, and *para*-methoxyphenylboronic acid). Attach the flask to a Schlenk line or a similar inert gas setup to allow for nitrogen purging.

To establish an inert atmosphere, flush the flask with nitrogen for 10–15 minutes, ensuring the flask is equipped with a rubber septum or a glass stopper to maintain the seal. If using a septum, insert a needle connected to the nitrogen line and a vent needle for purging. Remove the vent needle once the purging is complete.

Next, use a syringe to add the specified amounts of liquid reagents and solvent to the flask through the septum. Seal the septum securely after the addition. Place the flask in an oil bath or on a stirring hot plate set to the desired reaction temperature, typically 80 °C. The reaction mixture should be stirred at this temperature for the required duration, generally 4 hours.

After the reaction, the flask should be removed from the heat and allowed to cool to room temperature under an inert atmosphere. Once cooled, the reaction can be quenched by adding saturated ammonium chloride solution. The organic layer should be extracted and the sample analysed with an LC system as described in the main manuscript.

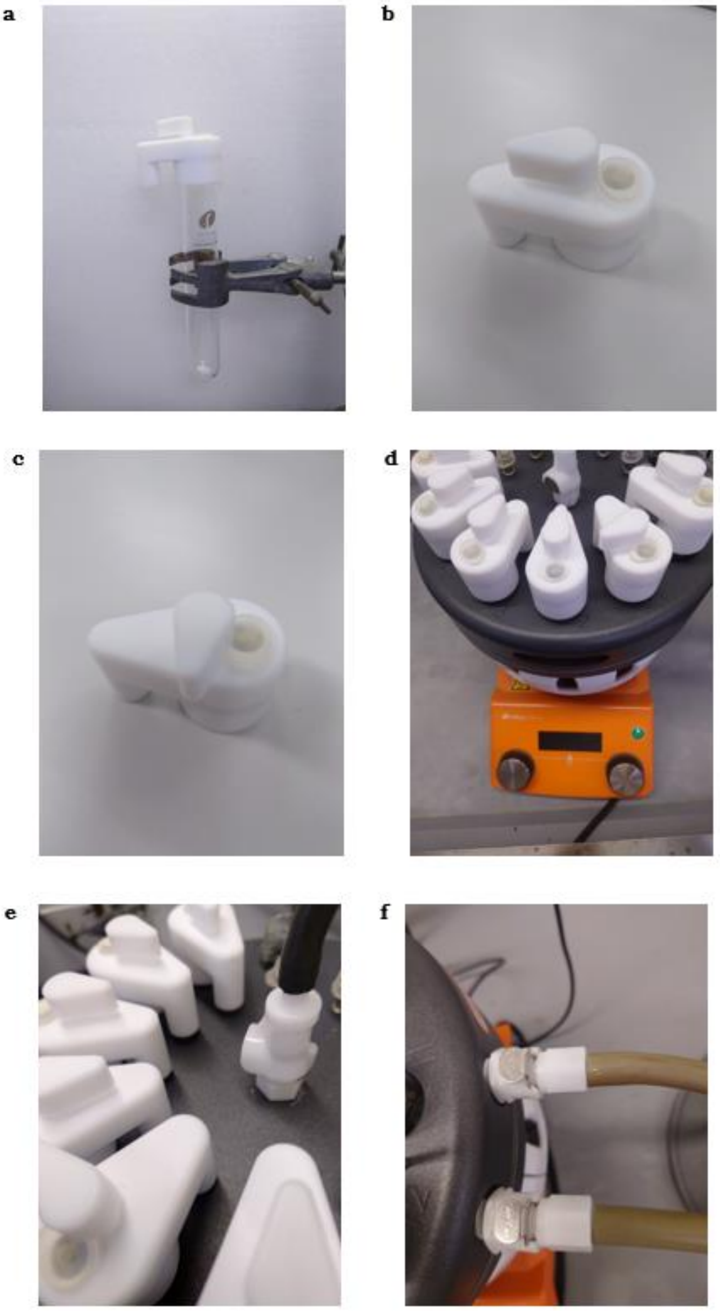

Figure S5: Implementation of a Radley's carousel for automated synthesis in this workflow. **a.** Individual reaction vessel equipped with magnetic flea. **b.** Lid of reaction vessel set open to nitrogen (parallel to gas flow) for dynamic purging **c.** Lid of reaction vessel set closed to inert gas (perpendicular to gas flow) with a septum for addition or reagents *via* syringe **d.** Reactor setup, including heating block and twelve reaction vessels. **e.** Connection to inert gas through central inlet. **f.** Connections to water for cooling.

The following software was used to implement this course:

- [MODDE Pro 13.0.1]
- Chemspeed Autosuite 2.4.12.0
- Chemspeed Crystal Solid Handling Robot 2.16.6.6

The following hardware was used to implement this course:

- Chemspeed SWING Automated Synthesis Platform
- Chemspeed Crystal Powderdose Solid Handling Robot
- Agilent 1100 LC System

Risk Assessment

The following risk assessment was created in-house for the experimental campaign executed in the main manuscript:

Department of Chemistry Experiment Risk Assessment V2.0

Please complete a risk assessment and gain approval before commencing activities. Numbers in square brackets (e.g., [2]) refer to the relevant MSDS section where information may be found.

Reaction scheme (include all reagents, solvents, reaction conditions, products and potential by-products, e.g., from side reactions, to match table below)

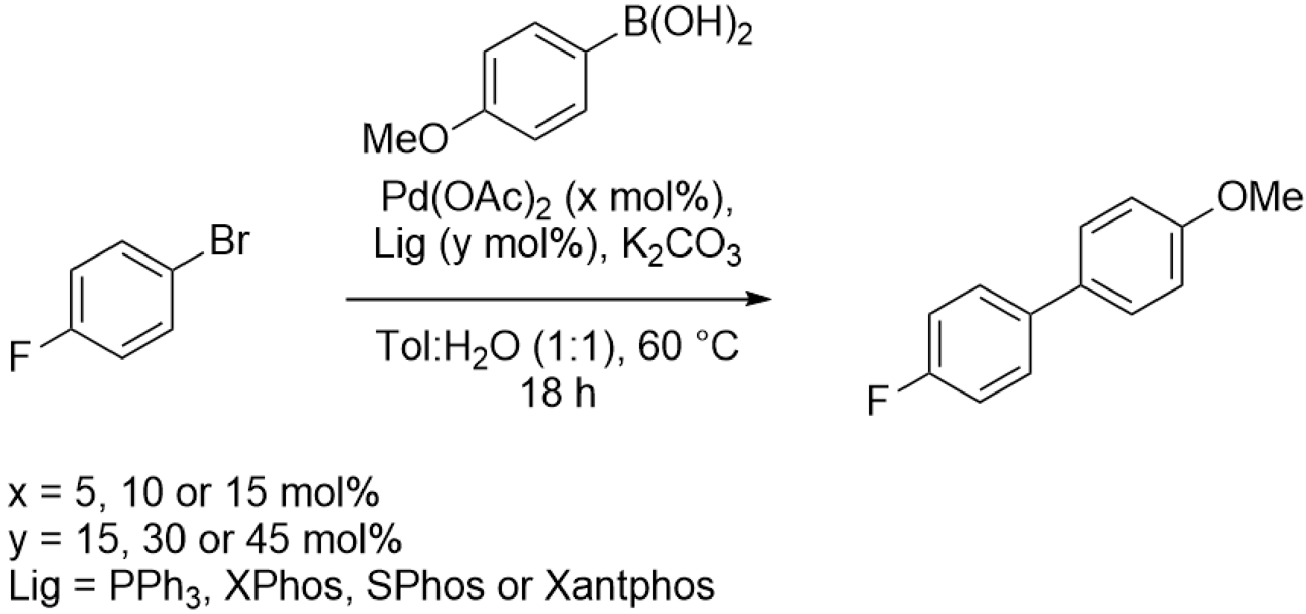

Suzuki-Miyaura Cross-Coupling reaction using various Pd and ligand loadings in order to optimize conversion to the desired product.

Chemical and Material Hazards

Include solvents, materials involved in work-up, and products including any anticipated by-products below, and in the scheme above.

Compound. Provide chemical name or label descriptor as indicated in scheme above. Hyperlink (ctrl+k) to MSDS and provide CAS if known. For prepared compounds, also provide notebook reference.	Quantity Include units.	State	Hazard codes and phrases if known [2]; list any exposure limits or health monitoring required [8]; consequences of exposure; any instabilities under ambient conditions [10]. Indicate if risk posed to pregnant or breastfeeding workers.
1-Bromo-4-fluorobenzene (**4**)<br>460-00-4	22 µL	Liquid	H226 - Flammable liquid and vapor<br>H315 - Causes skin irritation<br>H319 - Causes serious eye irritation<br><br>Contains no substances with occupational exposure limit values.<br><br>Reactivity:<br>Vapor/air-mixtures are explosive at intense warming.<br><br>Chemical stability:<br>The product is chemically stable under standard ambient conditions (room temperature) .<br><br>Possibility of hazardous reactions:<br>Violent reactions possible with:<br>Strong oxidizing agents<br>Strong acids<br>strong alkalis<br>various plastics<br><br>Conditions to avoid:<br>Heating.<br><br>Incompatible materials:<br>No data available
4-Methoxyphenyl boronic acid (**1**)<br>5720-07-0	33.4 mg	Solid	H315 - Causes skin irritation<br>H319 - Causes serious eye irritation<br>H335 - May cause respiratory irritation<br><br>Contains no substances with occupational exposure limit values.

			Reactivity:<br>The following applies in general to flammable organic substances and mixtures:<br>In correspondingly fine distribution, when whirled up a dust explosion potential may generally be assumed.<br><br>Chemical stability:<br>The product is chemically stable under standard ambient conditions (room temperature) .<br><br>Possibility of hazardous reactions:<br>Violent reactions possible with:<br>Strong oxidizing agents<br>Strong reducing agents<br>Strong acids<br>Strong alkalis<br><br>Conditions to avoid:<br>no information available<br><br>Incompatible materials:<br>No data available
$Pd(OAc)_2$<br>3375-31-3	2.2 - 6.7 mg	Solid	H317 - May cause an allergic skin reaction<br>H318 - Causes serious eye damage<br>H410 - Very toxic to aquatic life with long lasting effects<br>Contains no substances with occupational exposure limit values.<br><br>Reactivity:<br>The following applies in general to flammable organic substances and mixtures: in correspondingly fine distribution, when whirled up a dust explosion potential may generally be assumed.<br><br>Chemical stability:<br>The product is chemically stable under standard ambient conditions (room temperature) .<br><br>Possibility of hazardous reactions:<br>increased reactivity with:<br>Strong oxidizing agents<br>Metals

			Conditions to avoid:<br>no information available<br><br>Incompatible materials:<br>Aluminum,<br>Mild steel,<br>Strong oxidizing agents
PPh_3<br>603-35-0	7.9 - 23.6 mg	Solid	H302 - Harmful if swallowed<br>H317 - May cause an allergic skin reaction<br>H318 - Causes serious eye damage<br>H372 - Causes damage to organs (CNS, PNS) through prolonged or repeated exposure if inhaled<br><br>Contains no substances with occupational exposure limit values.<br><br>Reactivity:<br>Forms explosive mixtures with air on intense heating. A range from approx. 15 Kelvin below the flash point is to be rated as critical.<br><br>The following applies in general to flammable organic substances and mixtures:<br>in correspondingly fine distribution,<br>when whirled up a dust explosion potential may generally be assumed.<br>Chemical stability:<br>The product is chemically stable under standard ambient conditions (room temperature) .<br><br>Possibility of hazardous reactions:<br>Violent reactions possible with:<br>Oxidizing agents<br>Strong acids<br><br>Conditions to avoid:<br>Strong heating.<br><br>Incompatible materials:<br>No data available
XPhos<br>564483-18-7	14.3 - 43 mg	Solid	Not a hazardous substance or mixture.

SPhos<br>657408-07-6	12.3 - 37 mg	Solid	Not a hazardous substance or mixture.
Xantphos<br>161265-03-8	17.4 - 52 mg	Solid	H315 - Causes skin irritation<br>H319 - Causes serious eye irritation<br>H335 - May cause respiratory irritation<br><br>Contains no substances with occupational exposure limit values.<br><br>Reactivity:<br>The following applies in general to flammable organic substances and mixtures: in correspondingly fine distribution, when whirled up a dust explosion potential may generally be assumed.<br><br>Chemical stability:<br>The product is chemically stable under standard ambient conditions (room temperature) .<br><br>Possibility of hazardous reactions:<br>Violent reactions possible with:<br>Strong oxidizing agents<br><br>Conditions to avoid:<br>no information available<br><br>Incompatible materials:<br>No data available
K_2CO_3<br>584-08-7	60.8 mg	Solid	H315 - Causes skin irritation<br>H319 - Causes serious eye irritation<br>H335 - May cause respiratory irritation<br><br>Contains no substances with occupational exposure limit values.<br><br>Reactivity:<br>No data available<br><br>Chemical stability:<br>The product is chemically stable under standard ambient conditions (room temperature) .<br><br>Possibility of hazardous reactions:

			Violent reactions possible with:<br>Generates dangerous gases or fumes in contact with:<br>acids powdered alkaline earth metals halogen-halogen compounds<br><br>Risk of explosion with:<br>Halogenated<br>Hydrocarbon<br>Calcium<br>Carbon<br>With heat<br><br>Conditions to avoid:<br>Exposure to moisture<br><br>Incompatible materials:<br>no information available
Trimethoxybenzene (**2**)<br>621-23-8	16.8 mg	Solid	H302 - Harmful if swallowed<br><br>Contains no substances with occupational exposure limit values.<br><br>Reactivity:<br>The following applies in general to flammable organic substances and mixtures:<br>in correspondingly fine distribution, when whirled up a dust explosion potential may generally be assumed.<br><br>Chemical stability:<br>The product is chemically stable under standard ambient conditions (room temperature) .<br><br>Possibility of hazardous reactions:<br>Violent reactions possible with:<br>Strong oxidizing agents<br><br>Conditions to avoid:<br>no information available<br>Incompatible materials:<br>No data available
Toluene (**3**)<br>108-88-3	1.0 mL	Liquid	H225 - Highly flammable liquid and vapor<br>H3014 - May be fatal if inhaled and enters airways<br>H315 - Causes skin irritation

			H336 - May cause drowsiness and dizziness<br>H361d - Suspected of damaging unborn child<br>H373 - May cause damage to organs (CNS) through prolonged or repeated exposure if inhaled<br>H412 - Harmful to aquatic life with long lasting effects<br><br>Exposure limit: 192 mg/m3<br>In 450 m3 lab = 86.3 g = 99.7 mL<br>Way above what will be used<br><br>Reactivity:<br>Vapors may form explosive mixture with air.<br><br>Chemical stability:<br>The product is chemically stable under standard ambient conditions (room temperature).<br><br>Possibility of hazardous reactions:<br>Risk of explosion with:<br>fuming sulfuric acid<br>Nitric acid<br>silver perchlorates<br>nitrogen dioxide<br>nonmetallic halides<br>halogen-halogen compounds<br>uranium hexafluoride<br>organic nitro compounds<br><br>Violent reactions possible with:<br>Strong acids<br>Strong oxidizing agents<br>sulfur<br>with Heat.<br><br>Conditions to avoid:<br>Warming.<br><br>Incompatible materials:<br>No data available
Water<br>7732-18-5	1.0 mL	Liquid	Not a hazardous substance or mixture

Assessor: I confirm that less hazardous materials have been considered and, if applicable, have explained below why they are not being used in this case.	•
Details	

Assessor: I confirm that the risks of scaling up this particular experiment leading to runaway reaction or explosion have been considered (if applicable). If such risks are applicable, I have explained and provided the relevant notebook entry for the smaller scale experiment below.	•
Details	

Select all that apply to the chemical hazards listed above.

• Explosive	• Flammable	• Strong oxidizer	• Gas cylinder	• Corrosive
• Acutely toxic	• Irritant (skin, stench, etc.)	• Health hazard	• Environmental hazard	

Primary Containment and Control Measures

Provide additional details and considerations in the space provided.

Primary containment:	Other: give details	Chemspeed iSYNTH
Other control measures:	Other: give details	Chemspeed main platform

Assessor: I confirm to display an overnight safety card for all unattended or overnight reactions.	•

Techniques or Standard Methods Employed

Include purification and work up steps, and any characterization / spectroscopic methods.

Some techniques may require additional formal training.

Step	Techniques. Hyperlink (ctrl+k) to relevant SOP(s) or training record.
Preparation of solvents	Glassware handling<br>Use of needles and syringes

Solid dispensing (Chemspeed)	Crystal Powder Dose Export-Import SOP.docx
HPLC (Chemspeed)	HPLC SOP.docx

Authorization: I confirm that the person undertaking this activity and/or handling these substance(s) have been provided with information and training in its use, as far as practicable, and that relevant SOPs have been read and understood.	•

Physical hazards: (temperature, sharps, glassware, pressure, etc.); who may be harmed	Sharps hazard from needles

Procedure and Mitigations

Use the boxes at the end of the form if more space is required for detailed information.

Possible exposure routes:	Dermal, Inhalation	
Level and duration of exposure:	Very low	
Who could be exposed:	JJW; CSH	
Waste disposal: (Include details of any reagent quenching)	Standard waste streams	Give details
Spill clean-up method (consider all foreseeable scenarios):	Standard: give details	Give details
First aid measures [4]:	Standard: give details	1-Bromo-4-fluorobenzene:<br>General advice:<br>Show this material safety data sheet to the doctor in attendance.<br><br>If inhaled:<br>After inhalation: fresh air.<br><br>In case of skin contact:<br>In case of skin contact: Take off immediately all contaminated clothing.

		Rinse skin with water/ shower.<br><br>In case of eye contact:<br>After eye contact: rinse out with plenty of water.<br>Call in ophthalmologist.<br>Remove contact lenses.<br><br>If swallowed:<br>After swallowing: immediately make victim drink water (two glasses at most).<br>Consult a physician.<br><br>4-Methoxyphenyl boronic acid:<br>General advice:<br>Show this material safety data sheet to the doctor in attendance.<br><br>If inhaled:<br>After inhalation: fresh air.<br><br>In case of skin contact:<br>Take off immediately all contaminated clothing.<br>Rinse skin with water/ shower.<br><br>In case of eye contact:<br>After eye contact: rinse out with plenty of water.<br>Call in ophthalmologist.<br>Remove contact lenses.<br><br>If swallowed:<br>After swallowing: immediately make victim drink water (two glasses at most).<br>Consult a physician.<br><br>$Pd(OAc)_2$:<br>General Advice:<br>Show this material safety data sheet to the doctor in attendance.<br><br>If inhaled:<br>After inhalation: fresh air.<br><br>In case of skin contact:<br>Take off immediately all contaminated clothing.<br>Rinse skin with water/ shower.<br>Consult a physician.<br><br>In case of eye contact:<br>After eye contact: rinse out with plenty of water.<br>Immediately call-in ophthalmologist.<br>Remove contact lenses.<br><br>If swallowed:

		After swallowing: immediately make victim drink water (two glasses at most).<br>Consult a physician.<br><br>PPh_3:<br>General advice:<br>Show this material safety data sheet to the doctor in attendance.<br><br>If inhaled:<br>After inhalation: fresh air.<br>Call the physician.<br><br>In case of skin contact:<br>Take off immediately all contaminated clothing.<br>Rinse skin with water/ shower.<br>Consult a physician.<br><br>In case of eye contact:<br>After eye contact: rinse out with plenty of water.<br>Immediately call in ophthalmologist.<br>Remove contact lenses.<br><br>If swallowed:<br>After swallowing: immediately make victim drink water (two glasses at most).<br>Consult a physician.<br><br>XPhos:<br>If inhaled:<br>After inhalation: fresh air.<br><br>In case of skin contact:<br>Take off immediately all contaminated clothing.<br>Rinse skin with water/ shower.<br><br>In case of eye contact:<br>After eye contact: rinse out with plenty of water.<br>Remove contact lenses.<br><br>If swallowed:<br>After swallowing: make victim drink water (two glasses at most).<br>Consult doctor if feeling unwell.<br><br>SPhos:<br>If inhaled:<br>After inhalation: fresh air.<br>In case of skin contact In case of skin contact:<br>Take off immediately all contaminated clothing.<br>Rinse skin with water/ shower.<br><br>In case of eye contact:

		After eye contact: rinse out with plenty of water.<br>Remove contact lenses.<br><br>If swallowed:<br>After swallowing: make victim drink water (two glasses at most).<br>Consult doctor if feeling unwell.<br><br>Xantphos:<br>General advice:<br>Show this material safety data sheet to the doctor in attendance.<br><br>If inhaled:<br>After inhalation: fresh air.<br><br>In case of skin contact:<br>Take off immediately all contaminated clothing.<br>Rinse skin with water/ shower.<br><br>In case of eye contact:<br>After eye contact: rinse out with plenty of water.<br>Call in ophthalmologist.<br>Remove contact lenses.<br><br>If swallowed:<br>After swallowing: immediately make victim drink water (two glasses at most).<br>Consult a physician.<br><br>1,3,5-Trimethoxybenzene:<br>General advice:<br>Show this material safety data sheet to the doctor in attendance.<br><br>If inhaled:<br>After inhalation: fresh air.<br><br>In case of skin contact:<br>Take off immediately all contaminated clothing.<br>Rinse skin with water/ shower.<br><br>In case of eye contact:<br>After eye contact: rinse out with plenty of water.<br>Remove contact lenses.<br><br>If swallowed:<br>After swallowing: immediately make victim drink water (two glasses at most).<br>Consult a physician.<br><br><br>K_2CO_3

		General advice:<br>Show this material safety data sheet to the doctor in attendance.<br><br>If inhaled:<br>After inhalation: fresh air.<br><br>In case of skin contact In case of skin contact:<br>Take off immediately all contaminated clothing.<br>Rinse skin with water/ shower.<br><br>In case of eye contact:<br>After eye contact: rinse out with plenty of water.<br>Call in ophthalmologist.<br>Remove contact lenses.<br><br>If swallowed:<br>After swallowing: immediately make victim drink water (two glasses at most).<br>Consult a physician.<br><br>Toluene:<br>General advice:<br>Show this material safety data sheet to the doctor in attendance.<br><br>If inhaled:<br>After inhalation: fresh air.<br>Call the physician.<br><br>In case of skin contact:<br>Take off immediately all contaminated clothing.<br>Rinse skin with water/ shower.<br>Consult a physician.<br><br>In case of eye contact:<br>After eye contact: rinse out with plenty of water.<br>Call in ophthalmologist.<br>Remove contact lenses.<br><br>If swallowed:<br>After swallowing: caution if victim vomits. Risk of aspiration! Keep airways free.<br>Pulmonary failure possible after aspiration of vomit.<br>Call a physician immediately.<br><br>Water:<br>General advice:<br>No hazards which require special first aid measures.
Fire extinguisher [5]:	CO_2	Give details

(consider all substances in use)		
Any additional hazards to pregnant or breastfeeding workers?	Yes: give details	Toluene: H361d - May cause damage to unborn child
Is any health surveillance required?	No	
Is a DSEAR risk assessment required?	No	
Other emergency details:		

Exceptional PPE

Eye protectio n:	Safety glasses	Give details
Lab coat:	Cotton	Give details
Gloves:	Disposable nitrile	Toluene - 0 min
Other PPE:	N/A	

Additional Information

Use these boxes to add further details as required that are not sufficiently covered above.

E.g.,

- Detail any specific quenching procedures required to make waste mixtures safe before disposal
- Detail any specific firefighting procedures [5]: combine extinguisher types, considering substances in use together (e.g. foam extinguisher for all substances)
- Detail any procedures for transportation to an external site [14]
- Detail any specialized first aid measures [4]
- Detail any biological agents and their control
- Detail any radioactivity involved
- Detail any lasers in use that require training
- Detail any possible degradation to a more dangerous state for compounds in use

If the fire alarm sounds:

- You should switch off heating equipment (but not cooling), provided it is safe for you to do so, and pull-down fume hood sashes.
- Leave the building as soon as possible, using the nearest exit. Do not stop to collect personal belongings and do not use the lift.
- From E114 (lab) and E102 (write-up area), the nearest fire assembly point is the car park by Alcuin, beyond the liquid nitrogen tank. Wait here for further instruction.
- Do not re-enter the building until you are told it is safe to do so.

In case of fire in our lab:

- Shout for help and then, if appropriate and you have been trained, extinguish the fire with the correct extinguisher. If it is a small fire, e.g. solvent in a beaker – this can be extinguished simply by placing a watch glass over the beaker.
- It is possible that the fume hood fire trace will trigger, releasing a jet of powder or carbon dioxide to attempt to extinguish the flames.
- If a fire gets worse, or if you are in any doubt, set off the alarm and leave immediately, turning off gas and heating equipment.

Red fire alarm trigger points can be found in the stairwell lobby (E109 - on the way to the E-block stairs), immediately outside the lab door by the lift (D124), and in the corridor beyond IJSF office (D113).

Reporting of Fires:

- If you are involved in a fire incident where the fire alarm is triggered you must find a representative of the Departmental Safety Team as soon as possible and report it in full.
- The departmental operations manager should be consulted promptly following the use of any fire extinguishers or the triggering of a fire-trace.
- An online incident form should be filled in for any fires, even if no injuries are sustained: https://www.york.ac.uk/univ/mis/cfm/accidents/.

No transportation to an external site indicated.

No specialized first aid measures indicated.

No biological agents indicated.

No radioactivity indicated.

No use of lasers indicated.
Product formed has unknown hazards and toxicity - assume hazardous
For general safety procedures, consult E114 Safety protocol: Safety Protocol - E114 - v1.0

Assessor: I confirm that the foreseeable risks will be controlled and minimized as far as is practicable where they cannot be removed.	•

Maternity/Breastfeeding

Hazard codes of particular concern during pregnancy or breastfeeding:

- H340, H341, H350, H350i, H351, H360, H360D, H360FD, H360Fd, H360Df, H361, H361d, H361fd, H362, H370, H371

Substances of particular concern during pregnancy or breastfeeding:

- Mercury and mercury derivatives
- Antimitotic (cytotoxic) drugs
- Carbon monoxide
- Chemical agents of known and dangerous percutaneous absorption
- Lead and lead derivatives
- Preparations labelled on the basis of Directive 1999/45/EC
- Chemical agents and industrial processes in Annex 1 to Directive 90/394/EEC (Control of Carcinogenic Substances)

Additional Files

In additional files (or screenshots), please find:

i. PowerPoint presentation with a guide to setting up the automation required for automation campaign detailed in the main manuscript
ii. Crystal PowderDose Files
iii. Chemspeed SWING files
iv. MODDE files

Initial Course Developments (footnote i, main manuscript)

Our initial attempt to address the lack of coverage of automation techniques and data-led approaches to reaction optimization in University curricula was *via* design and delivery of a third-year undergraduate mini-project. These research-led projects are a key part of the integrated masters undergraduate (MChem) programme delivered at the University of York (Department of Chemistry). They consist of a structured and closely supervised group research project spread over eight days in teaching labs with further time made available for data analysis and interpretation. These projects are carried out towards the end of the students' third-year with the aim of introducing them to the independent research skills required for their final year 4 research projects. The project designed for this work focused on the development and optimization of a DOE model in a Suzuki-Miyaura coupling reaction space with the aim of finding greener alternatives to common cross-coupling solvents whilst maintaining reaction performance.

Students screened an initial set of twelve solvents with a range of cross coupling partners and analyzed reaction outcomes relative to a range of solvent descriptors: the Kamlet-Abboud-Taft $\alpha/\beta/\pi^*$ system, Hansen solubility parameters, dielectric constant and dipole moment. Sigma-profiles generated from COSMO-RS modelling were also offered as qualitative solvent descriptors, however they were ultimately not used. From this data, students were tasked with choosing the solvent descriptor that was responsible for the largest degree of variance in reaction outcomes and utilizing it in a fractional factorial design DOE alongside base pKa and reaction temperature. Practically executing these reactions allowed students to develop predictive models on this reaction space. This model was tested across a wide range of cross coupling partners selected to offer a wide range of synthetic interest (**Figure S6**).

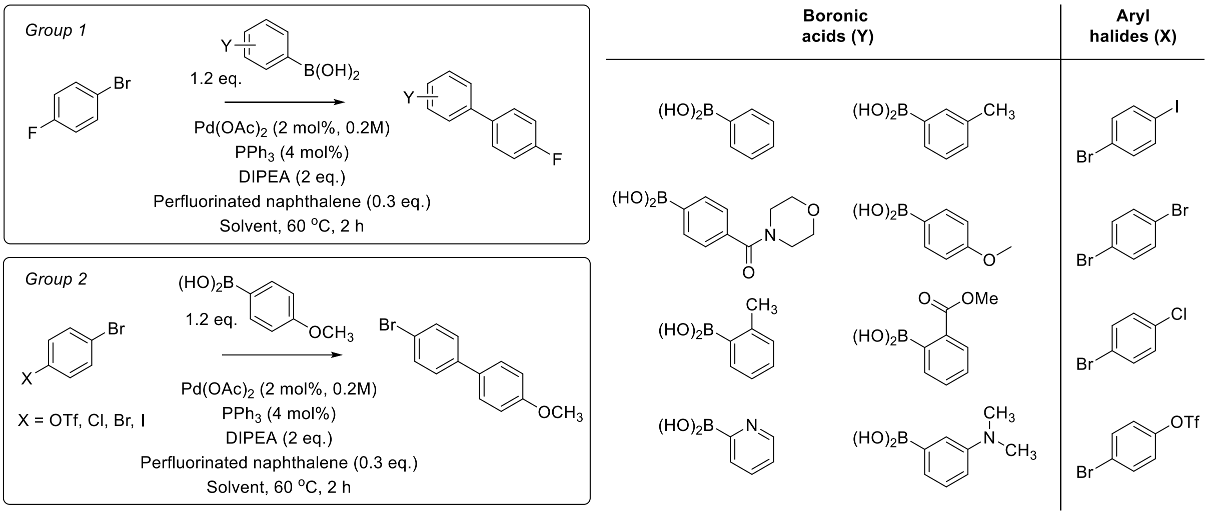

Figure S6: The range of substrates provided to students for testing, offering a wide range of synthetic challenge.

Reactions were carried out by students using Radley's Carousel reactors, introducing students to parallel reaction screening without the need for training and access to a fully automated reaction platform. Lower cost alternatives based on septum vials, multi-site heating blocks and nitrogen lines were also tested during development of the project and proved to be viable lower-cost options for this chemistry.

Through a process of pedagogical reflection on student and peer feedback, we made the decision to reformulate the laboratory course into the postgraduate workshop outlined in the main body of this work. The most significant issue with the design of the project was that the solvent effect on the chosen set of reactions proved far more complex than initial experiments had indicated. It was difficult, therefore, for students to draw meaningful correlations between any one solvent parameter and reaction outcome (within the project timeframe available: 8 days). Students generally felt that the project had enhanced their level of skills and knowledge, that all group members had felt able to participate and that the workload was appropriate. We also received feedback that the overall research question was unclear and that 8 days

was not long enough to tackle such an open-ended project to the students' satisfaction. Despite good engagement from students and clear improvements in their technical skills and research output as the project progressed, the vast volume of experiments and relatively complex data analysis requirements made this a challenging topic for students' first experience of research.

From the above experiences, it was concluded that it would be more appropriate to introduce and explore the concepts of data-led reaction optimization at a later stage in chemists' research careers and to do so *via* lecture and demonstration approaches. This allowed further emphasis to be placed on conceptual understanding of HTE experimentation and data analysis, without the requirement for learners to run large amounts of experiments themselves. Synthetic chemists working at a postgraduate level also tend to have experience optimizing reactions *via* traditional One Factor At a Time (OFAT) methods. These conclusions ultimately led us to develop the postgraduate course which is the main focus of the present manuscript.

References

1 Niwa, T.; Ochiai, H.; Watanabe, Y.; Hosoya, T. Ni/Cu-Catalyzed Defluoroborylation of Fluoroarenes for Diverse C-F Bond Functionalizations. *J. Am. Chem. Soc.* **2015**, *137*, 14313-14318. DOI: 10.1021/jacs.5b10119.
